# Supplementary material for: Analysis of Flavonoid Metabolites in Citrus reticulata ‘Chachi’ at Different Collection Stages Using UPLC-ESI-MS/MS
Source: Foods. 2023 Oct 28;12(21):3945. doi: 10.3390/foods12213945 (PMC10648965; doi:10.3390/foods12213945)
Supplement: Supplementary file 1 [file foods-12-03945-s001.zip › foods-2631544-supplementary.pdf]

Figure S1: Calibration diagrams used for the quantitative analysis of metabolites. The integration correction results are shown for random samples, and the x-axis represents retention time.

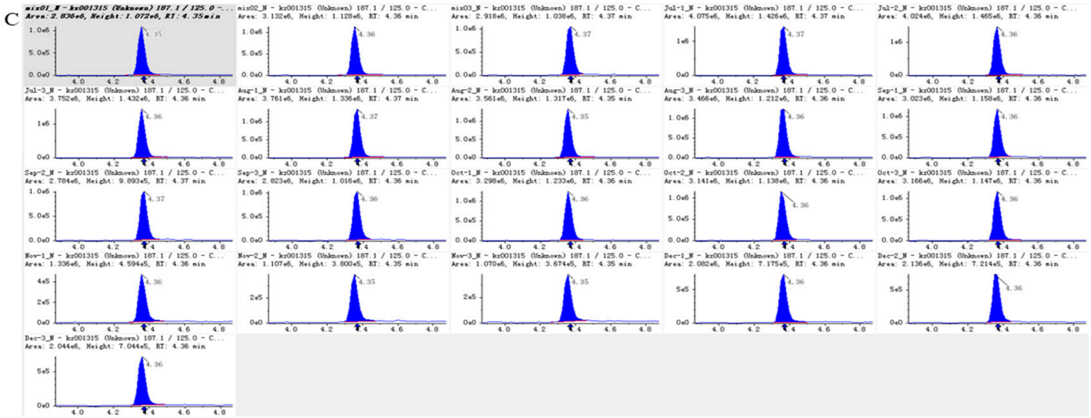

Table S1: Flavonoid compounds in CRCP at different picking stages.

No.	Compound	Molecular Weight (Da)	Class	Relative content (%)					
				Jul	Aug	Sep	Oct	Nov	Dec
**1**	Apigenin	270.05	Flavones	0.05±0.00	0.05±0.00	0.08±0.00	0.06±0.00	0.03±0.00	0.02±0.00
**2**	Chrysoeriol	300.06	Flavones	0.23±0.02	0.47±0.01	0.73±0.03	0.50±0.01	0.56±0.01	0.46±0.00
**3**	Apigenin 7-O-neohesperidoside	578.16	Flavones	0.23±0.01	0.14±0.01	0.15±0.00	0.17±0.01	0.25±0.01	0.25±0.00
**4**	Tricin	330.00	Flavones	1.17±0.07	2.55±0.05	2.74±0.01	2.43±0.00	1.93±0.04	1.16±0.03
**5**	Velutin	314.08	Flavones	0.33±0.03	4.32±0.27	6.53±0.28	4.84±0.27	5.13±0.19	2.84±0.25
**6**	O-methylnaringenin C-pentoside	418.13	Flavones	0.66±0.04	0.39±0.03	0.44±0.02	0.67±0.03	0.69±0.04	0.84±0.03
**7**	Diosmetin	300.06	Flavones	0.26±0.02	0.53±0.01	0.80±0.02	0.55±0.01	0.62±0.02	0.51±0.01
**8**	5,7,4'-Trimethoxyflavone	312.10	Flavones	0.10±0.01	0.09±0.00	0.07±0.00	0.07±0.00	0.10±0.00	0.08±0.00
**9**	5,7-Dihydroxy-3',4',5'-trimethoxyflavone	344.09	Flavones	0.22±0.00	0.24±0.01	0.21±0.01	0.21±0.00	0.16±0.00	0.14±0.00
**10**	7-Hydroxy-3,5,6,8-tetramethoxyflavone	358.11	Flavones	0.07±0.01	0.06±0.00	0.04±0.00	0.05±0.00	0.05±0.00	0.04±0.00

**11**	5-Hydroxy-6,7,8,3',4'-pentamethoxyflavone	388.12	Flavones	0.11±0.01	0.09±0.01	0.08±0.01	0.11±0.00	0.06±0.01	0.04±0.01
**12**	Apigenin 5-O-glucoside	432.11	Flavones	2.07±0.56	1.18±0.08	1.26±0.16	1.37±0.20	1.64±0.10	2.34±0.08
**13**	Chrysoeriol-5-O-hexoside	462.12	Flavones	0.07±0.01	0.06±0.02	0.04±0.00	0.05±0.00	0.07±0.01	0.06±0.00
**14**	Diosmetin-6-C-glucoside	462.12	Flavones	0.21±0.01	0.15±0.01	0.12±0.01	0.15±0.00	0.19±0.02	0.16±0.01
**15**	Tricin O-saccharic acid	522.10	Flavones	0.06±0.00	0.06±0.00	0.16±0.01	0.16±0.00	0.24±0.01	0.20±0.00
**16**	Isorhoifolin	578.16	Flavones	0.23±0.01	0.15±0.01	0.15±0.00	0.18±0.00	0.26±0.01	0.26±0.00
**17**	Apigenin 6,8-C-diglucoside	594.16	Flavones	1.51±0.03	1.31±0.01	1.09±0.02	1.10±0.01	1.58±0.03	1.29±0.01
**18**	Diosmin	608.17	Flavones	0.28±0.04	0.34±0.04	0.35±0.05	0.31±0.02	0.93±0.11	0.72±0.09
**19**	Luteolin-6,8-di-C-glucoside	610.15	Flavones	0.8±0.05	0.75±0.03	0.56±0.01	0.59±0.03	0.89±0.01	0.74±0.01
**20**	Chrysoeriol-O-sinapoylhexoside	668.17	Flavones	0.07±0.01	0.20±0.02	0.33±0.02	0.43±0.01	0.42±0.06	0.55±0.01
**21**	Apigenin 6-C-hexosyl-8-C-hexosyl-O-hexoside	756.21	Flavones	0.01±0.00	0.01±0.00	0.02±0.00	0.02±0.00	0.03±0.00	0.02±0.00

**22**	Monohydroxy-trimethoxyflavone	328.09	Flavones	0.38±0.01	0.26±0.01	0.16±0.00	0.20±0.00	0.17±0.00	0.15±0.00
**23**	6-Hydroxyluteolin 5-glucoside	464.10	Flavones	0.35±0.02	0.31±0.02	0.35±0.05	0.44±0.06	0.35±0.09	0.60±0.00
**24**	6-hydroxy-5,7,4'-trimethoxyflavone	328.09	Flavones	0.76±0.01	0.87±0.01	0.89±0.02	0.97±0.01	1.18±0.01	0.93±0.00
**25**	4'-hydroxy-5,6,7-trimethoxyflavone	328.09	Flavones	0.30±0.02	0.22±0.01	0.19±0.01	0.20±0.01	0.14±0.00	0.15±0.01
**26**	Tetramethylluteolin	342.11	Flavones	0.17±0.01	0.14±0.00	0.13±0.00	0.14±0.00	0.17±0.00	0.15±0.00
**27**	7,8-Dihydroxy-5,6,4'-trimethoxyflavone	344.09	Flavones	0.29±0.01	0.53±0.01	0.52±0.01	0.48±0.01	0.46±0.01	0.29±0.00
**28**	2,4,4'-trihydroxychalcone	256.07	Flavones	0.00±0.00	0.00±0.00	0.00±0.00	0.00±0.00	0.00±0.00	0.00±0.00
**29**	Daidzein-hexoside-xyloside	548.15	Flavones	0.00±0.00	0.00±0.00	0.00±0.00	0.00±0.00	0.00±0.00	0.00±0.00
**30**	7-O-methylnaringenin	286.08	Flavones	0.01±0.00	0.01±0.00	0.00±0.00	0.00±0.00	0.00±0.00	0.00±0.00

**31**	Tricin-O-rutinoside	638.18	Flavones	2.55±0.18	2.22±0.23	1.54±0.16	1.62±0.04	2.77±0.23	1.99±0.18
**32**	Chrysoeriol-feruloylglu	638.16	Flavones	0.18±0.02	0.23±0.01	0.19±0.01	0.15±0.01	0.09±0.01	0.14±0.01
**33**	Isosaponarin	594.16	Flavones	0.41±0.01	0.59±0.03	0.57±0.02	0.60±0.01	0.49±0.04	0.35±0.00
**34**	Mearnsetin	332.05	Flavones	0.01±0.00	0.01±0.00	0.01±0.00	0.02±0.00	0.02±0.00	0.03±0.00
**35**	Chrysin-O-glucoside	416.11	Flavones	0.00±0.00	0.00±0.00	0.00±0.00	0.00±0.00	0.00±0.00	0.01±0.00
**36**	Eupatorin	344.09	Flavones	0.11±0.01	0.11±0.00	0.08±0.00	0.09±0.00	0.08±0.00	0.07±0.00
**37**	5-hydroxy-3,7,4'-trimethoxyflavone	328.09	Flavones	0.01±0.00	0.04±0.00	0.05±0.00	0.04±0.00	0.06±0.00	0.03±0.00
**38**	5-Hydroxy-6,7,3',4'-tetramethoxyflavone	358.11	Flavones	0.34±0.00	0.37±0.00	0.28±0.01	0.30±0.01	0.43±0.00	0.36±0.00
**39**	5-Hydroxy-3,6,7,4'-Tetramethoxyflavone	358.11	Flavones	2.76±0.00	2.54±0.03	2.40±0.05	2.92±0.03	1.93±0.00	1.51±0.01
**40**	Tilianin	446.12	Flavones	0.00±0.00	0.00±0.00	0.00±0.00	0.00±0.00	0.00±0.00	0.00±0.00
**41**	Acacetin-7-O-galactoside	446.12	Flavones	0.00±0.00	0.00±0.00	0.00±0.00	0.00±0.00	0.00±0.00	0.00±0.00
**42**	Diosmetin-7-O-galactoside	462.12	Flavones	0.33±0.04	0.45±0.04	0.39±0.01	0.35±0.05	0.29±0.00	0.36±0.03

**43**	Patuletin-3-O-β-D-glucopyranoside	494.11	Flavones	0.20±0.00	0.13±0.01	0.09±0.00	0.07±0.00	0.05±0.00	0.04±0.01
**44**	Acacetin-7-O-rutinoside	592.18	Flavones	0.19±0.02	0.21±0.01	0.27±0.01	0.18±0.01	0.82±0.02	0.90±0.02
**45**	Luteolin-7-O-rutinoside	594.16	Flavones	1.33±0.05	1.10±0.05	0.98±0.04	1.00±0.06	1.84±0.09	1.61±0.03
**46**	mlyricetin 3-O-B-D-glucopyranoside	464.10	Flavones	1.80±0.07	1.68±0.01	1.38±0.01	1.32±0.06	0.81±0.05	1.35±0.09
**47**	Apigenin-7-O-(2''-O-p-Coumarylglucoside)	578.14	Flavones	0.22±0.00	0.14±0.01	0.15±0.01	0.18±0.00	0.26±0.01	0.26±0.01
**48**	(2R)-Pinocembrin-7-neohesperidoside	564.18	Flavones	0.41±0.00	0.30±0.00	0.26±0.00	0.40±0.01	0.71±0.01	0.73±0.01
**49**	Tamarixetin 3-O-rutinoside	624.17	Flavones	0.08±0.01	0.10±0.01	0.13±0.01	0.14±0.00	0.33±0.01	0.29±0.01
**50**	Apigenin-7-O-β-D-glucoside	432.11	Flavones	0.05±0.00	0.05±0.02	0.10±0.01	0.07±0.00	0.10±0.02	0.07±0.02
**51**	Luteolin-caffeoyl-O-rhamnoside	594.14	Flavones	0.24±0.01	0.16±0.01	0.24±0.01	0.29±0.01	0.46±0.00	0.59±0.01
**52**	Dihydroxy-dimethoxyflavone	314.08	Flavones	0.94±0.02	0.80±0.01	0.86±0.02	1.00±0.02	0.43±0.01	0.32±0.00

**53**	5,7,8,4'-Tetramethoxyflavone	342.11	Flavones	1.91±0.12	1.52±0.02	1.24±0.02	1.32±0.02	1.67±0.04	1.58±0.00
**54**	tetramethoxyflavanone	344.13	Flavones	0.26±0.00	0.25±0.01	0.30±0.00	0.32±0.01	0.12±0.00	0.09±0.00
**55**	Isosinensetin	372.12	Flavones	1.54±0.09	1.31±0.01	0.97±0.03	1.02±0.01	1.48±0.03	1.23±0.03
**56**	Sinensetin	372.12	Flavones	0.09±0.01	0.07±0.00	0.05±0.00	0.06±0.00	0.08±0.00	0.07±0.00
**57**	Nobiletin	402.13	Flavones	0.61±0.02	0.5±0.01	0.43±0.01	0.48±0.02	0.64±0.01	0.61±0.01
**58**	Dihydroxy-dimethoxyflavone-O-glucoside	476.13	Flavones	0.26±0.01	0.54±0.04	1.04±0.04	0.72±0.02	1.02±0.02	0.74±0.02
**59**	Tamarixetin-malonylhexoside	564.11	Flavones	0.12±0.00	0.15±0.01	0.20±0.01	0.19±0.01	0.04±0.01	0.08±0.01
**60**	Neodiosmin	608.17	Flavones	1.38±0.20	1.63±0.22	1.70±0.23	1.54±0.12	4.42±0.52	3.53±0.42
**61**	O-methylChrysoeriol 5-O-hexoside	476.13	Flavones	0.04±0.00	0.13±0.01	0.18±0.00	0.12±0.00	0.19±0.01	0.14±0.00
**62**	Quercetin 3-O-galactoside	464.10	Flavonols	2.14±0.07	2.03±0.02	1.66±0.02	1.56±0.07	1.02±0.05	1.65±0.05
**63**	Dihydroquercetin	304.06	Flavonols	0.01±0.00	0.02±0.00	0.02±0.00	0.02±0.00	0.01±0.00	0.01±0.00
**64**	Kaempferol 3-O-galactoside	448.10	Flavonols	0.07±0.00	0.05±0.00	0.04±0.00	0.02±0	0.06±0.02	0.05±0.01
**65**	Kaempferol 3-O-glucoside	448.10	Flavonols	0.00±0.00	0.01±0.00	0.00±0.00	0.00±0.00	0.00±0.00	0.00±0.00
**66**	Kaempferol 3-O-rutinoside	594.16	Flavonols	0.08±0.00	0.07±0.00	0.07±0.00	0.07±0.01	0.12±0.01	0.12±0.00

**67**	Kaempferol 7-O-rhamnoside	432.11	Flavonols	0.44±0.01	0.28±0.01	0.38±0.00	0.43±0.01	0.66±0.02	0.95±0.03
**68**	Isotrifoliin	464.10	Flavonols	0.34±0.03	0.30±0.03	0.34±0.02	0.47±0.03	0.38±0.01	0.57±0.04
**69**	Quercetin 4'-O-glucoside	464.10	Flavonols	0.13±0.01	0.09±0.01	0.09±0.01	0.11±0.01	0.15±0.01	0.16±0.01
**70**	Troxerutin	346.25	Flavonols	0.12±0.08	0.13±0.08	0.13±0.09	0.11±0.1	0.16±0.13	0.24±0.18
**71**	Di-O-methylquercetin	330.10	Flavonols	0.85±0.03	0.85±0.02	0.62±0.01	0.65±0.03	0.75±0.04	1.36±0.08
**72**	Kaempferol	286.05	Flavonols	0.00±0.00	0.00±0.00	0.00±0.00	0.00±0.00	0.00±0.00	0.00±0.00
**73**	Quercetin	302.04	Flavonols	0.02±0.00	0.02±0.00	0.02±0.00	0.02±0.00	0.04±0.00	0.03±0.00
**74**	Isorhamnetin	316.06	Flavonols	0.03±0.00	0.16±0.01	0.52±0.04	0.29±0.03	0.27±0.02	0.14±0.01
**75**	Syringetin	346.07	Flavonols	0.32±0.03	0.74±0.03	0.85±0.03	0.65±0.04	0.36±0.00	0.19±0.01
**76**	3,5,6,7,8,3',4'-Heptamethoxyflavone	432.14	Flavonols	0.69±0.05	0.58±0.01	0.43±0.01	0.49±0.00	0.58±0.01	0.47±0.00
**77**	Isorhamnetin-O-acetyl-hexoside	520.12	Flavonols	0.02±0.00	0.03±0.00	0.06±0.00	0.06±0.00	0.01±0.00	0.03±0.00
**78**	Tiliroside	594.14	Flavonols	0.24±0.01	0.16±0.01	0.24±0.00	0.30±0.01	0.47±0.01	0.60±0.01
**79**	Quercetin-3-O-robinobioside	610.15	Flavonols	4.16±0.22	3.90±0.14	3.87±0.03	3.08±0.19	1.48±0.12	3.52±0.15
**80**	Rutin	610.15	Flavonols	3.72±0.22	3.72±0.05	3.81±0.10	3.24±0.18	1.95±0.04	4.00±0.29
**81**	6-Hydroxykaempferol-7,6-O-Diglucoside	626.15	Flavonols	0.17±0.02	0.32±0.03	0.37±0.02	0.38±0.02	0.42±0.02	0.61±0.03

**82**	Limocitrin-3-O-(3-hydroxy-3-methylglutarate)-glucoside	652.16	Flavonols	1.39±0.09	0.99±0.05	0.81±0.03	0.55±0.01	0.17±0.02	0.18±0.01
**83**	isorhamnetin-O-glucoside-O-glucoside	640.16	Flavonols	0.03±0.00	0.08±0.01	0.18±0.01	0.15±0.00	0.21±0.01	0.16±0.00
**84**	Quercetin-O-rutinoside-O-rhamnoside	756.21	Flavonols	0.07±0.00	0.11±0.03	0.13±0.03	0.10±0.01	0.10±0.01	0.11±0.01
**85**	Isorhamnetin-O-rutinoside-O-rhamnoside	770.23	Flavonols	0.04±0.01	0.08±0.01	0.09±0.00	0.12±0.02	0.10±0.01	0.10±0.01
**86**	syringetin-O-rutinoside-O-glucoside	816.23	Flavonols	0.06±0.00	0.04±0.00	0.03±0.00	0.04±0.00	0.07±0.01	0.04±0.00
**87**	Quercetin-O-Hexoside-O-Hexoside-O-Pentoside	758.19	Flavonols	0.00±0.00	0.01±0.00	0.01±0.00	0.00±0.00	0.01±0.00	0.01±0.00
**88**	Syringetin-O-glucoside	508.12	Flavonols	0.49±0.04	0.44±0.07	0.56±0.06	0.58±0.05	0.14±0.03	0.13±0.02
**89**	Kaempferol-3-O-(6''-acetyl)-glucoside	490.11	Flavonols	0.16±0.01	0.18±0.00	0.10±0.00	0.12±0.01	0.08±0.00	0.10±0.00

**90**	Quercetin-3-O-(6''-O-acetyl)-galactoside	506.11	Flavonols	0.07±0.00	0.07±0.00	0.05±0.00	0.04±0.01	0.06±0.01	0.09±0.01
**91**	Kaempferol-3-O-(6''-malonyl)-glucoside	534.10	Flavonols	0.00±0.00	0.01±0.00	0.01±0.00	0.00±0.00	0.01±0.00	0.00±0.00
**92**	Kaempferol-3-O-(6''-malonyl)-galactoside	534.10	Flavonols	0.00±0.00	0.01±0.00	0.01±0.00	0.00±0.00	0.01±0.00	0.01±0.00
**93**	Quercetin-3-O-(6''-O-malonyl)-galactoside	550.10	Flavonols	0.11±0.01	0.10±0.00	0.07±0.00	0.07±0.01	0.07±0.00	0.08±0.01
**94**	Limocitrin	346.07	Flavonols	0.13±0.01	0.25±0.01	0.29±0.02	0.22±0.02	0.12±0.01	0.07±0.01
**95**	Limocitrin-O-arabinoside	478.11	Flavonols	0.12±0.01	0.08±0.01	0.05±0.01	0.05±0.01	0.01±0.00	0.01±0.00
**96**	Limocitrin 3-rhamnoside	492.13	Flavonols	0.30±0.02	0.27±0.02	0.23±0.03	0.29±0.02	0.21±0.03	0.19±0.03
**97**	Limocitrin 3-galactoside	508.12	Flavonols	0.49±0.06	0.45±0.05	0.55±0.05	0.55±0.05	0.10±0.01	0.12±0.00
**98**	Limocitrin 3-sophoroside	670.17	Flavonols	0.08±0.01	0.10±0.01	0.11±0.00	0.13±0.01	0.32±0.02	0.18±0.01
**99**	Limocitrin 3,7-diglucoside	670.17	Flavonols	0.09±0.01	0.10±0.00	0.12±0.01	0.13±0.00	0.32±0.01	0.19±0.01
**100**	Limocitrin-O-Hexoside-O-	802.22	Flavonols	0.00±0.00	0.00±0.00	0.00±0.00	0.00±0.00	0.00±0.00	0.00±0.00

	Hexoside-O-Pentoside								
**101**	Isorhamnetin-O-Hexoside-O-Hexoside-O-Rhamnoside	786.22	Flavonols	0.04±0.00	0.05±0.00	0.07±0.00	0.07±0.00	0.08±0.00	0.08±0.00
**102**	Isorhamnetin-7-O-glucoside	478.11	Flavonols	1.04±0.10	0.83±0.06	0.7±0.02	0.67±0.04	0.44±0.05	0.45±0.05
**103**	Isorhamnetin-O-Hexoside-O-Pentoside	610.15	Flavonols	0.00±0.00	0.00±0.00	0.00±0.00	0.00±0.00	0.00±0.00	0.00±0.00
**104**	Quercetin-7-O-(6'-O-malonyl)-β-D-glucoside	550.10	Flavonols	0.01±0.00	0.01±0.00	0.01±0.00	0.01±0.00	0.01±0.00	0.02±0.00
**105**	Isorhamnetin-3-O-rutinoside (Narcissin)	624.17	Flavonols	0.19±0.01	0.21±0.00	0.15±0.01	0.14±0.00	0.12±0.00	0.14±0.00
**106**	Kaempferol-3-O-neohesperidoside	594.16	Flavonols	0.01±0.00	0.01±0.00	0.00±0.00	0.00±0.00	0.00±0.00	0.00±0.00
**107**	Isorhamnetin O-malonylglucoside	564.11	Flavonols	0.12±0.01	0.16±0.01	0.20±0.01	0.19±0.01	0.04±0.01	0.07±0.00
**108**	Isorhamnetin 3-O-β-D-Glucoside	478.11	Flavonols	1.07±0.11	0.81±0.05	0.68±0.02	0.69±0.03	0.45±0.03	0.46±0.02
**109**	Rhamnetin 3-O-β-D-Glucoside	478.11	Flavonols	0.81±0.01	0.81±0.05	0.59±0.05	0.56±0.03	0.42±0.03	0.54±0.04

**110**	Quercetin-O-sinapoylglucosid-O-glucoside	832.21	Flavonols	0.00±0.00	0.00±0.00	0.00±0.00	0.00±0.00	0.00±0.00	0.00±0.00
**111**	Quercetin-O-sinapoylglucosid-O-glucoside-rhamnoside	978.26	Flavonols	0.00±0.00	0.00±0.00	0.00±0.00	0.00±0.00	0.00±0.00	0.00±0.00
**112**	Dihydrokaempferol-O-glucoside	450.12	Flavonols	0.11±0.01	0.08±0.00	0.06±0.00	0.10±0.00	0.09±0.00	0.08±0.00
**113**	Tangeretin	372.12	Flavonols	0.25±0.01	0.20±0.00	0.19±0.00	0.21±0.01	0.26±0.01	0.26±0.00
**114**	Natsudaidain 3-O-(3-hydroxy-3-methylglutarate)-glucoside)	724.22	Flavonols	1.18±0.04	0.50±0.01	0.46±0.02	0.53±0.01	0.28±0.01	0.27±0.00
**115**	Kaempferol 3-O-[2-O-β-D-xylose-6-O-a-L-rhamnose]-β-D-glucoside	726.20	Flavonols	0.03±0.00	0.02±0.00	0.01±0.00	0.01±0.00	0.01±0.00	0.01±0.00
**116**	Vitexin	432.11	Flavone C-glycosides	3.14±0.06	3.12±0.11	2.62±0.07	2.70±0.06	1.73±0.12	1.59±0.05
**117**	Isohemiphloin	434.12	Flavone C-glycosides	0.04±0.00	0.04±0.00	0.06±0.01	0.05±0.00	0.03±0.00	0.02±0.00
**118**	Isoorientin	448.10	Flavone C-glycosides	2.58±0.19	3.34±0.23	2.93±0.14	2.56±0.06	1.71±0.11	2.31±0.12

**119**	Orientin	448.10	Flavone C-glycosides	3.27±0.20	3.30±0.15	2.60±0.06	2.71±0.16	1.87±0.10	2.32±0.16
**120**	Apigenin-6-C-glucose-8-xylcose	564.15	Flavone C-glycosides	1.30±0.08	1.77±0.07	1.54±0.02	1.58±0.10	1.43±0.07	1.04±0.03
**121**	6-C-Hexosyl luteolin O-pentoside	580.14	Flavone C-glycosides	0.19±0.01	0.29±0.03	0.25±0.00	0.25±0.01	0.24±0.02	0.30±0.00
**122**	Isovitexin 7-O-glucoside	594.16	Flavone C-glycosides	0.39±0.01	0.35±0.01	0.30±0.01	0.34±0.01	0.42±0.01	0.37±0.01
**123**	Chrysoeriol-6,8-di-C-glucoside	624.17	Flavone C-glycosides	0.43±0.01	0.38±0.00	0.31±0.00	0.33±0.01	0.45±0.01	0.40±0.00
**124**	Genistein 8-C-glucoside	432.11	Flavone C-glycosides	3.27±0.07	3.12±0.08	2.65±0.10	2.70±0.10	1.74±0.13	1.57±0.04
**125**	5,3',4'-trihydroxy-6,7-dimethoxyflavone-8-C-β-D-glucoside	492.13	Flavone C-glycosides	0.12±0.00	0.08±0.01	0.05±0.00	0.06±0.00	0.04±0.00	0.04±0.00
**126**	5,7,4'-trihydroxy-8-methoxyflavone-6-C-[β-D-Xylosyl-(1-2)]-β-D-glucoside	594.16	Flavone C-glycosides	0.91±0.06	2.12±0.04	1.94±0.05	1.92±0.06	1.53±0.20	1.86±0.12
**127**	5,7,4'-trihydroxy-6-	594.16	Flavone C-glycosides	0.91±0.11	2.02±0.09	1.92±0.07	1.93±0.14	1.56±0.14	1.86±0.06

	methoxyflavone-8-C-[β-D-Xylosyl-(1-2)]-β-D-glucoside								
**128**	5,7,4'-trihydroxy-6-methoxyflavone-8-C-[β-D-glucosyl-(1-2)]-β-D-glucoside	624.17	Flavone C-glycosides	3.01±0.02	2.61±0.11	2.53±0.01	2.30±0.01	3.61±0.08	3.29±0.08
**129**	Vitexin-2-O-D-glucopyranoside	594.16	Flavone C-glycosides	0.80±0.01	0.69±0.02	0.58±0.02	0.60±0.01	0.88±0.04	0.73±0.03
**130**	Narirutin	580.18	Flavanone	0.67±0.04	0.36±0.02	0.40±0.02	0.60±0.02	0.62±0.04	0.76±0.04
**131**	Neohesperidin	610.19	Flavanone	4.80±0.13	4.58±0.17	5.28±0.07	5.92±0.24	8.70±0.06	8.06±0.07
**132**	Poncirin	594.20	Flavanone	0.20±0.01	0.13±0.01	0.20±0.00	0.25±0.01	0.38±0.01	0.48±0.01
**133**	Naringenin 7-O-neohesperidoside	580.18	Flavanone	0.74±0.03	0.4±0.01	0.44±0.00	0.66±0.01	0.66±0.02	0.83±0.02
**134**	Hesperetin O-malonylhexoside	550.20	Flavanone	0.20±0.01	0.09±0.00	0.06±0.00	0.09±0.01	0.13±0.01	0.14±0.01
**135**	Naringenin O-malonylhexoside	520.12	Flavanone	0.10±0.00	0.05±0.00	0.03±0.00	0.03±0.00	0.03±0.00	0.03±0.00
**136**	Prunin	434.12	Flavanone	0.28±0.00	0.14±0.01	0.17±0.00	0.29±0.02	0.12±0.00	0.14±0.01
**137**	Narirutin 4'-glucoside	742.23	Flavanone	0.05±0.00	0.03±0.00	0.06±0.00	0.10±0.01	0.10±0.00	0.14±0.00
**138**	Natsudaidain	418.13	Flavanone	7.22±0.11	5.48±0.02	4.07±0.04	5.36±0.02	2.63±0.09	2.56±0.08
**139**	Biochanin A	284.07	Isoflavones	0.20±0.01	0.52±0.02	0.85±0.03	0.62±0.01	0.53±0.01	0.31±0.01

**140**	Genistein	270.05	Isoflavones	0.05±0.00	0.05±0.00	0.08±0.00	0.06±0.00	0.03±0.00	0.02±0.00
**141**	5,7,3',4'-tetrahydroxyisoflavone	286.05	Isoflavones	0.39±0.04	0.24±0.01	0.32±0.02	0.37±0.02	0.59±0.05	0.82±0.07
**142**	Genistein 7-O-Glucoside	432.11	Isoflavones	1.94±0.14	1.28±0.09	1.26±0.12	1.74±0.16	1.87±0.10	2.16±0.37
**143**	Formononetin	268.07	Isoflavones	0.00±0.00	0.00±0.00	0.00±0.00	0.00±0.00	0.00±0.00	0.00±0.00
**144**	Prunetin	284.07	Isoflavones	0.2±0.01	0.52±0.01	0.84±0.02	0.60±0.02	0.54±0.02	0.31±0.01
**145**	Formononetin 7-O-β-D-glycoside	430.13	Isoflavones	0.01±0.00	0.01±0.00	0.00±0.00	0.01±0.00	0.01±0.00	0.00±0.00
**146**	Glycitein	284.07	Isoflavones	0.00±0.01	0.00±0.00	0.01±0.02	0.00±0.00	0.03±0.05	0.00±0.00
**147**	Pinocembrin	256.07	Dihydroflavone	0.00±0.00	0.00±0.00	0.00±0.00	0.00±0.00	0.00±0.00	0.00±0.00
**148**	Isosakuranetin	286.08	Dihydroflavone	0.01±0.00	0.01±0.00	0.02±0.00	0.02±0.00	0.01±0.00	0.01±0.00
**149**	Eriodictyol	288.06	Dihydroflavone	0.08±0.00	0.06±0.00	0.09±0.00	0.08±0.00	0.01±0.00	0.03±0.00
**150**	5,6,7,8,3',4'-Hexamethoxyflavanone	404.15	Dihydroflavone	1.21±0.05	0.87±0.04	0.68±0.00	0.93±0.01	0.38±0.00	0.45±0.01
**151**	Naringenin-7-O-glucoside	434.12	Dihydroflavone	0.04±0.00	0.02±0.00	0.03±0.00	0.05±0.00	0.02±0.00	0.02±0.00
**152**	Eriodictyol 7-O-glucoside	450.12	Dihydroflavone	0.13±0.01	0.08±0.01	0.07±0.00	0.09±0.00	0.15±0.01	0.13±0.02
**153**	3',5-Dihydroxy-4',6,7-	346.11	Dihydroflavone	0.2±0.01	0.16±0.00	0.11±0.00	0.11±0.00	0.13±0.00	0.09±0.00

	Trimethoxyflavanone								
**154**	5-Hydroxy-6,7,3',4'-tetramethoxyflavanone	360.12	Dihydroflavone	0.94±0.02	0.82±0.01	0.57±0.00	0.70±0.01	0.82±0.02	0.62±0.01
**155**	3',4',5,6,7-pentamethoxyflavanone	374.14	Dihydroflavone	1.67±0.15	1.30±0.04	0.96±0.03	1.10±0.02	1.31±0.03	1.05±0.01
**156**	Casticin	374.10	Dihydroflavone	0.00±0.00	0.00±0.00	0.00±0.00	0.00±0.00	0.00±0.00	0.00±0.00
**157**	Naringenin	272.07	Dihydroflavone	0.24±0.01	0.19±0.03	0.31±0.01	0.31±0.02	0.05±0.00	0.08±0.00
**158**	Hesperetin	302.08	Dihydroflavone	0.87±0.01	0.78±0.02	2.42±0.08	1.86±0.03	0.33±0.01	0.95±0.02
**159**	5,7,3',4',5'-Pentamethoxyflavanone	374.14	Dihydroflavone	0.32±0.01	0.24±0.00	0.22±0.01	0.18±0.00	0.04±0.00	0.05±0.00
**160**	Hesperidin	610.19	Dihydroflavone	4.49±0.25	4.25±0.24	4.92±0.06	5.53±0.22	8.14±0.05	7.62±0.18
**161**	Astilbin	450.12	Dihydroflavonol	0.11±0.00	0.08±0.01	0.06±0.00	0.10±0.01	0.09±0.00	0.08±0.00
**162**	Hesperetin 5-O-glucoside	464.13	Dihydroflavonol	0.35±0.01	0.32±0.01	0.39±0.04	0.49±0.05	0.41±0.06	0.60±0.10
**163**	Dihydrokaempferol	288.06	Dihydroflavonol	0.00±0.00	0.00±0.00	0.00±0.00	0.00±0.00	0.00±0.00	0.00±0.00

**164**	7-O-Methyleriodictyol	302.08	Flavanols	0.01±0.00	0.00±0.00	0.00±0.00	0.00±0.00	0.00±0.00	0.00±0.00
**165**	Eriodictyol O-malonylhexoside	696.07	Flavanols	0.04±0.00	0.03±0.00	0.02±0.00	0.02±0.00	0.03±0.00	0.03±0.00
**166**	Gallocatechin-catechin	594.14	Flavanols	0.43±0.00	0.4±0.01	0.41±0.03	0.39±0.01	0.72±0.02	0.70±0.03
**167**	Phloretin	274.08	Chalcones	0.00±0.00	0.00±0.00	0.00±0.00	0.00±0.00	0.00±0.00	0.00±0.00
**168**	Phlorizin	436.14	Chalcones	0.02±0.00	0.01±0.00	0.01±0.00	0.01±0.00	0.01±0.00	0.01±0.00

Data are expressed as means ± standard deviation (n = 3).
